# For Someone, You Are the Whole World: Host-Specificity of *Salmonella enterica*

**DOI:** 10.3390/ijms241813670

**Published:** 2023-09-05

**Authors:** Anastasiya V. Merkushova, Anton E. Shikov, Anton A. Nizhnikov, Kirill S. Antonets

**Affiliations:** 1 Laboratory for Proteomics of Supra-Organismal Systems, All-Russia Research Institute for Agricultural Microbiology (ARRIAM), 196608 St. Petersburg, Russia; a.merkushova@arriam.ru (A.V.M.); a.shikov@arriam.ru (A.E.S.); a.nizhnikov@arriam.ru (A.A.N.); 2 Faculty of Biology, St. Petersburg State University (SPbSU), 199034 St. Petersburg, Russia

**Keywords:** *Salmonella enterica*, pangenome, host specificity, virulence, human infections

## Abstract

*Salmonella enterica* is a bacterial pathogen known to cause gastrointestinal infections in diverse hosts, including humans and animals. Despite extensive knowledge of virulence mechanisms, understanding the factors driving host specificity remains limited. In this study, we performed a comprehensive pangenome-wide analysis of *S. enterica* to identify potential loci determining preference towards certain hosts. We used a dataset of high-quality genome assemblies grouped into 300 reference clusters with a special focus on four host groups: humans, pigs, cattle, and birds. The reconstructed pangenome was shown to be open and enriched with the accessory component implying high genetic diversity. Notably, phylogenetic inferences did not correspond to the distribution of affected hosts, as large compact phylogenetic groups were absent. By performing a pangenome-wide association study, we identified potential host specificity determinants. These included multiple genes encoding proteins involved in distinct infection stages, e.g., secretion systems, surface structures, transporters, transcription regulators, etc. We also identified antibiotic resistance loci in host-adapted strains. Functional annotation corroborated the results obtained with significant enrichments related to stress response, antibiotic resistance, ion transport, and surface or extracellular localization. We suggested categorizing the revealed specificity factors into three main groups: pathogenesis, resistance to antibiotics, and propagation of mobile genetic elements (MGEs).

## 1. Introduction

Since its descendence from its common ancestor with *Escherichia coli*, the genus *Salmonella* became an invasive pathogen causing foodborne diseases in cold- and warm-blooded animals, including humans [1]. The genus consists of two species, namely, *S. bongori* and *S. enterica*, with the former encompassing six subspecies: *enterica* (I), *salamae* (II), *arizonae* (IIIa), *diarizonae* (IIIb), *houtenae* (IV), and *indica* (VI) [2]. Further serological-based subdivision according to the Kauffmann–White–Le Minor scheme has allowed for identifying more than 2600 individual serovars, with most of them (1500) pertaining to *S. enterica* subsp. *enterica* [3]. Nearly 50 highly infectious clinical serovars, by various estimates, relate to 115 to 179 million infection cases, 216,000–600,000 of which end up with a fatal outcome [4,5,6]. The concomitant annual losses reach up to USD 3.66 billion, as reported by the Centers for Disease Control and Prevention (CDC) [7]. In addition to human infections, frequent animal deaths caused by certain serovars such as Gallinarium and Dublin affecting poultry and cattle, respectively, intensify economic loss, which could be especially crucial for developing countries [8,9].

Due to epidemiological and clinical significance, an in-depth understanding of host specificity is important. Given that the infection process consists of multiple steps, almost all involved proteins could delineate successful colonization. Three main stages are distinguished: (i) colonization of the lumen, (ii) invasion of epithelial cells, and (iii) endocytosis or phagocytosis by host immune cells. Colonization requires attachment to host cells driven by surface structures and proteins such as fimbriae, adhesins, flagellin as well as antigenic lipopolysaccharides (LPS) [10]. In the second stage, the bacterium utilizes type III secretion systems (T3SS-1 and T3SS-2) to inject effector proteins provoking rearrangement of host cells’ cytoskeleton, with the concomitant formation of *Salmonella*-containing vacuole (SCV) via internalization [11,12]. The subsequent survival and propagation in macrophages commonly observed in the case of systemic infections are less studied; however, the role of other effectors translocated with T3SS-2 was reported [13].

Given that strains of *Salmonella* spp. have been isolated from nearly all vertebrates, e.g., fish, reptiles, amphibians, birds, and mammals, it is considered a so-called “universal pathogen” [14]. Despite such a characterization, known serovars differ sufficiently, being either bound to a single host or, conversely, exhibiting a broad host range. These two groups are commonly referred to as specialists and generalists, respectively [15]. Specialists, causing systemic yet predominantly symptomless infection with possible sporadic transmission to other hosts, are presented by serovars Dublin and Choleraesuis infecting cattle and pigs only, and are termed host-adapted [16]. Host-restricted serovars, in turn, affect one host exclusively, as shown by *S.* Typhi, a causative agent of typhoid fever in humans, or the bird-restricted *S.* Gallinarium [14]. Contrarily, serovars that are not bound to certain hosts comprise non-host-adapted strains, the most well-known of which are *S.* Typhimurium, *S.* Enteritidis, which is able to cause enteritidis in humans, poultry, cattle, pigs, and mice [17]. Therefore, farm animals become reservoirs posing a threat to global health due to potential outbreaks of enteric infection stemming, either from contact with animals or the consumption of contaminated food products such as meat and eggs [18].

Participants in the aforementioned stages contribute to host specificity, yet the exact mechanisms are poorly explored at present [19]. Multiple genomic and experimental studies show that both the presence and/or absence of certain genomic determinants contribute to host preference [19]. To mention a few, lower allelic diversity within genes encoding FimH adhesin was reported for host-restricted serovars such as *S.* Typhi and *S.* Choleraesuis, but not for broad-host-range *S.* Typhimurium and *S.* Enteritidis [20]. Serovar Rissen adapted to humans requires a full Typhi colonization factor (*tcf*) operon [21]. In contrast, the sparrow-restricted *S.* Typhimurium strain MpSTM, which is unable to infect mice, lost a virulence plasmid and T3SS-2 effectors [22]. A similar inactivation of the *speC* gene responsible for polyamine synthesis was observed in *S.* Typhi and *S.* Gallinarum adapted to humans and poultry [23]. Such genomic alterations in loci-encoding proteins involved in different infection stages include fimbriae, adhesion, membrane proteins, secretion systems, effectors, toxins, enzymes, and transcription factors, thereby implying that *Salmonella* isolates exhibit varying adaptive pathways leading to host restriction [19].

A promising approach to harnessing an intricate network of specificity determinants lies in pangenome analysis, which makes it possible to identify potential candidates prior to experimental verification. That being said, it seems surprising that the application of pangenomic tools in terms of host specificity remains limited. Primarily, research is focused on quite a few selected serovars. These include studying whole genome-based comparisons of isolates from diverse hosts, e.g., *S.* Derby and *S.* Mbandaka [24] or *S.* Gallinarum and *S.* Pullorum [25]. Other instances represent the analysis of differentiation between host-restricted and non-host-adapted strains [26,27], or characterizing bacterial populations from different sources within a single serovar [28]. At the same time, exploring the dataset of draft genomes belonging to multiple isolates enabled the revealing of common metabolical adaptations for serovars infecting the same group of hosts [29]. To this end, we performed a pangenome-wide data-driven study of the available high-quality genomes of *S. enterica,* aiming to identify host specificity factors.

## 2. Results

### 2.1. Dataset Preparation

**Figure 1:**
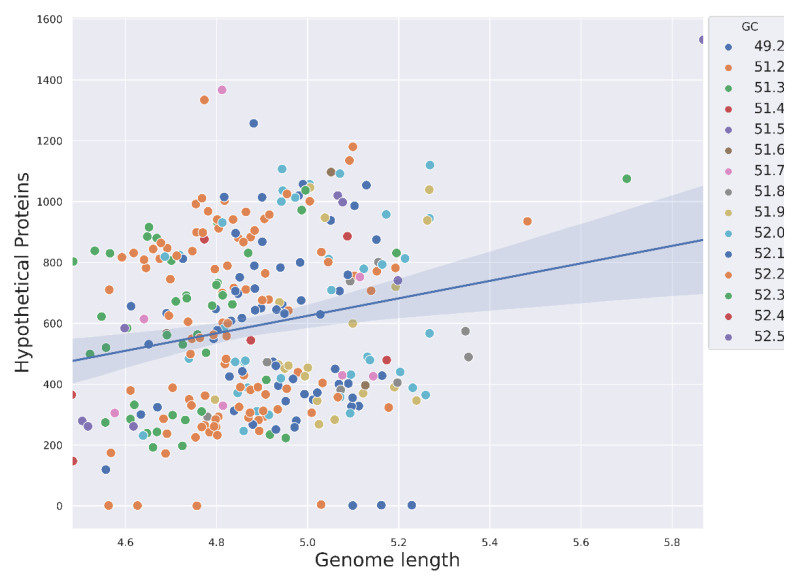
Genomic properties of the analyzed dataset comprising *S. enterica* genome assemblies. Shown is the relationship between genome length on the megabase scale and the number of hypothetical proteins in the assembly. The colors of points correspond to the mean GC content within genomes.

To reconstruct the pangenome, we first acquired 1598 genome assemblies of *Salmonella enterica* from the NCBI assembly database [30] (Table S1), resulting in 1055 assemblies passing the RefSeq database [31] filters (unusual length, contamination). The filtered dataset contained 126 serovars. To avoid bias in the association analysis, we removed assemblies with extensive similarity, leaving us with a final set of 300 assemblies after dereplication (Table S2), after which 110 serovars remained. The selected assemblies showed commensurable genome lengths, with an average of 4,891,720 b.p., and contained an average of 4589 CDS (Table S1). The average GC content of the genomes hovered around 52%. We have not revealed any remarkable relationships between CG content and genome size or the number of hypothetical proteins; however, the two latter properties, quite expectedly, positively correlated with each other (Figure 1). This similarity of genomic properties therefore allows us to consider the reliability of the dataset in terms of taxonomic relatedness.

### 2.2. Pangenome Reconstruction

**Figure 2:**
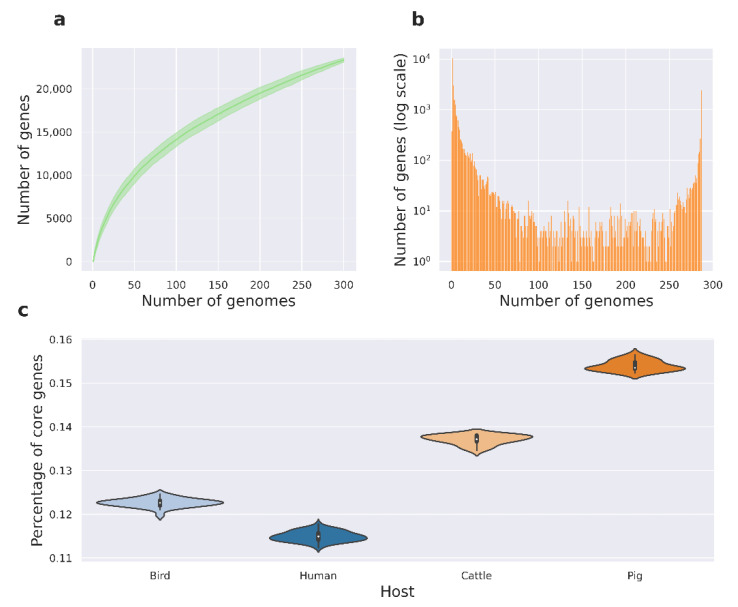
Main characteristics of the reconstructed pangenome. (**a**) The power–fit curve applying 1000 permutations depicting the number of pangenome clusters versus the number of genomes used for pangenome reconstruction. (**b**) Pangenome U-curve illustrating the frequency of pangenome clusters found in a certain number of genomes. (**c**) The ratio of the core component of the genome to the accessory component in the pangenome within the groups of assemblies attributed to different hosts.

Numbered lists can be added as follows: The pangenome reconstructed with Panaroo contained 27,845 gene clusters, 2796 of which represented core genes. According to the calculated alpha parameter reaching 0.56, the pangenome could be considered open. This openness was further confirmed by the saturation curve (Figure 2a), which did not reach a plateau. These findings may suggest high genetic variability within the genomes in the dataset, as supported by the substantial size of the accessory component of the genome. The pangenome U-curve (Figure 2b) exhibited a canonical shape without internal peaks, corroborating the taxonomic similarity between genomes and the absence of contamination in the pangenome.

We then classified chosen genomes according to the metadata of infected hosts, and attributed reference genomes to all the hosts from each assembly within the cluster. Metadata were acquired jointly from the PATRIC resource [32] and the BioSample [33] database. The dataset displayed the following distribution of hosts from which the strains were isolated: 139 unknown of origin, 80 humans, 32 birds, 20 pigs, 19 cattle, 5 reptiles, 2 sheep, 2 horses, and 1 canine, respectively (Table S2). We next focused on the groups associated with no less than 10 assemblies, and retained four host groups for the subsequent study. To assess the genome plasticity of assemblies attributed to diverse hosts, we calculated the ratios of the core component to the accessory component within pangenome clusters for each host category (Figure 2c), and found the values differed significantly (*p* < 0.01). Notably, assemblies associated with human hosts exhibited a lower number of core genes compared to those affecting other hosts. This observation might indicate that various groups of generalist strains infecting humans require larger accessory components in order to thrive in diverse host environments.

### 2.3. Host-Wise Characterization of Salmonella enterica Phylogeny

To reveal whether recombination affects the quality of reference phylogeny, we reconstructed two types of trees, namely, one type based on the sequences including regions subjected to recombination, and one type excluding them. According to topological comparisons, the quartet distance was 0.90, the average absolute distance measured by the Robinson–Fuld metric was 200, and the Cophenetic correlation reached 0.99, thus showing considerable congruence between trees. When examining trees’ quality, we found that the analyzed values were higher in the case of the tree devoid of recombination-prone regions, with the consistency index (CI) being equal to 0.25 and 0.33 and the retention index (RI) reaching 0.77 and 0.78 for trees with and without recombination signals, respectively. The Colles–Like balance index, however, indicated that the former tree was more balanced (4756 vs. 4915). Such an observation may not directly reflect the quality, as the most evident indicator (CI) showed higher levels of homoplasy if recombination occurs, which is consistent with current views [34]. Based on the results, we selected the recombination-free phylogeny for further analysis accordingly.

**Figure 3:**
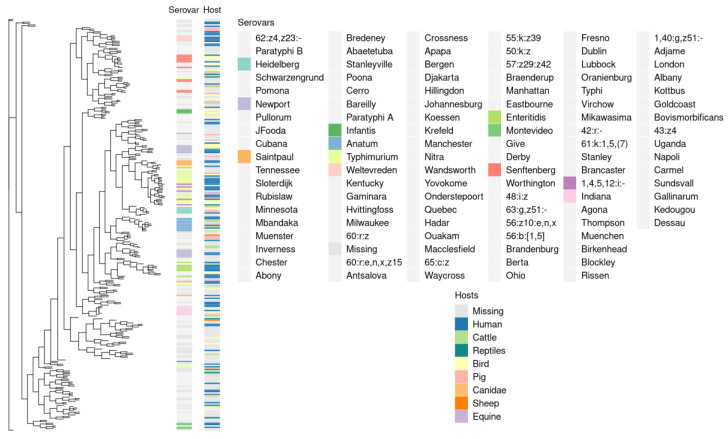
Core SNP-based reference phylogeny. The phylogeny was constructed using core SNPs (Single Nucleotide Polymorphism) excluding variants that occurred during recombination. The presented lengths of the tree branches are log-transformed. The adjacent heatmap illustrates the distribution of affected hosts and the serological attribution of the reference strains chosen for the built tree. Only the serovars to which at least 4 assemblies were attributed are colored for clarity.

To determine whether phylogenetic history reflects adaptation to hosts, we plotted the affected hosts as well as serovars positioned adjacent to the reference tree (Figure 3). While most of the serovars formed separate clades, there were no large phylogenetic clusters on the basis of host specificity. This observation supports the existent view of *S. enterica* as a universal pathogen capable of infecting a wide range of hosts. Such a broad host range backed by multiple adaptation routes is likely maintained by high genetic variation in terms of accessory genes’ composition.

### 2.4. Revealing Host Specificity Determinants and Known Virulence Factors

Using the pangenome-wide associating method (pan-GWAS) utilizing pyseer [35], we identified a total of 237 significant (Benjamini–Hochberg-adjusted *p*-value > 0.5) pangenomic clusters that were positively associated with certain hosts. We found no significant negative associations, while within the positive ones, 143 were related to cattle, 32 to avian, 61 to swine, and only 1 to human hosts, respectively (Table S5). The latter was presented by transposase, which gene was found in the assemblies affecting other hosts as well.

The most notable specificity determinants contributing to the specific infection of birds included genes encoding antibiotic resistance proteins, such as TetC, MFS efflux proteins, and ribokinase, as well as controlling the infectious process, such as the CIII protease inhibitor, the two-pore potassium channel, the Rhs protein, etc. The set of factors potentially determining preference towards pigs was linked with transcriptional regulation (*lexA*), pathogenesis (*sopE* effector, glycoside hydrolase), the transfer of mobile genetic elements (loci within insertions and prophages), and multiple components encoded by the *tra* operon (*traM*, *traN*, *traL*, *traO*). Finally, affecting cattle showed associations with resistance to heavy metals and antibiotics (*floR*, *merD*, *merB*, *merP*, *dsbC*), iron transport (Fe^3+^-siderophore permease), hemolysis (*hha*), and bacterial conjugation (*virD2*).

To determine which specificity determinants are known virulence factors, we analyzed the homology between protein sequences of predicted sets of specificity factors and those deposited in the VFDB database. We first analyzed all protein sequences in the entire pangenome and identified a total of 93,470 protein matches. No homologs were presented in the sets of specificity determinants associated with birds and humans, while, for other instances, only a negligible fraction of predicted factors corresponded to known virulence factors, with 9 out of 1695 (0.05%) and 11 out of 3665 (0.03%) found in groups associated with pigs and cattle, respectively.

### 2.5. Functional Annotation of Gene Groups

We then characterized functional patterns of multiple host specificity routes by performing an over-representation test considering functional annotations of host-wise significant positive associations (Table S7), using three ontologies from the Gene Orthology (GO) annotation system [36]: Biological Processes (BP), Molecular Functions (MF), and Cellular Components (CC).

**Figure 4:**
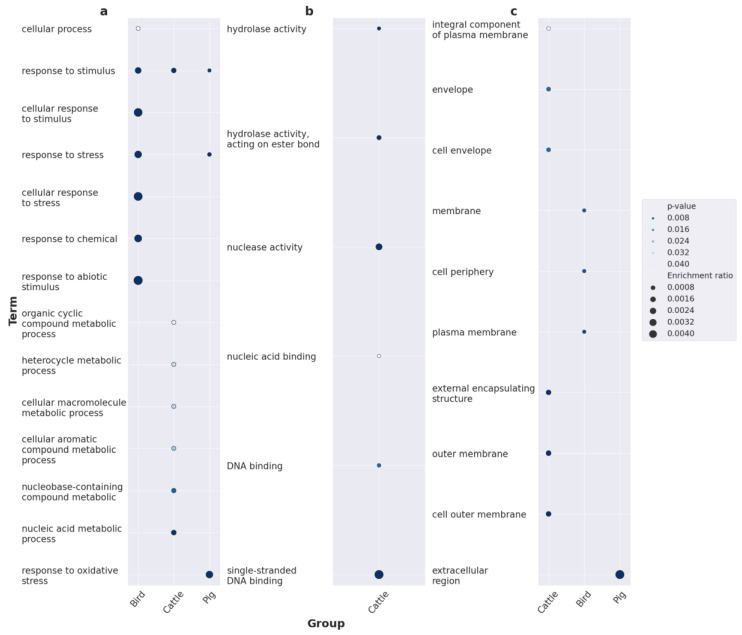
Functional annotation of host specificity factors with over-represented terms using the GO (Gene Ontology) annotation system. Shown are the results for the sets of pyseer-reported positive associations with certain hosts filtered according to Benjamini–Hochberg (BH) correction for multiple comparisons within the Biological Processes (**a**), Molecular Functions (**b**), and Cellular Components (**c**) ontologies. The dot size depicts the enrichment ratio, while the intensity of the color is proportional to *p*-values.

Within the BP category, infecting avian hosts appears to rely on response to various stimuli, particularly stress (Figure 4a). The genes associated with cattle included those exhibiting a wide range of enzymatic activities, especially connected to DNA metabolism: the synthesis of organic cyclic compounds, heterocycles, macromolecules, aromatic compounds, nucleobase-containing compounds, and nucleic acids. Somewhat similar to an avian infection, adaptation to swine hosts implies a reaction to stress and, notably, oxidative stress, possibly reflecting the evasion of the host’s immune response. We also found the unequal cellular localization of the putative specificity factors. Enrichments in the MF category gained results only for the set of factors providing adaptation to cattle. The inferences resembled and complemented the respective BP terms, namely, nucleic acid, DNA, and single-stranded DNA binding, as well as nuclease and hydrolase activity (Figure 4b). Proteins encoded by loci associated with cattle resided on the components of the outer membrane and envelope; those associated with birds were found in the cell periphery and plasma membrane, and those associated with pigs were found in the extracellular regions (Figure 4c).

To proceed with comparing well-studied virulence factors with potential host specificity determinants, we revealed significant GO enrichments accordingly. While there were some common GO terms, mostly linked with DNA metabolism, others fell into expected typical categories reflecting pathogenesis stages. Those encompassed protein binding, locomotion, biological regulation, transporter activity, locomotion, bacterial-type flagellum, organelles, and cell projection (Figure S1).

**Figure 5:**
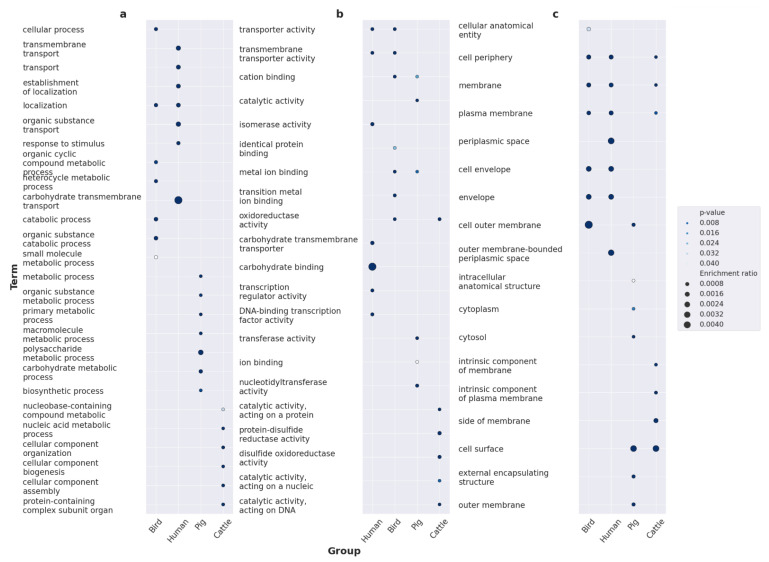
Functional annotation of host specificity factors with over-represented terms using the GO (Gene Ontology) annotation system. Shown are the results for the sets of pyseer-reported positive associations with certain hosts filtered according to raw *p*-values within the Biological Processes (**a**), Molecular Functions (**b**), and Cellular Components (**c**) ontologies. The dot size depicts the enrichment ratio, while the intensity of the color is proportional to *p*-values.

As the available data were limited, we further chose to focus on the general functional features by examining host specificity determinants, taking into account raw pyseer-generated *p*-values. The total number of studied pangenome clusters then increased to 1867, with 633 of them associated with pigs, 608 with cattle, 545 with birds, and 285 with humans. We have not obtained multiple novel insights in terms of biological processes due to the over-general functional terms reported. Nevertheless, it could be inferred that human pathogenesis actively involves membrane transport (Figure 5b). The extended dataset enabled enough statistical power to yield significant enrichments in the MF ontology. Plenty of annotations regarding ion and metal transport highlight their importance during pathogenic processes during the course of avian infection (Figure 5b). Adaptation to cattle, in its turn, implied catalytic activities, ranging from oxidoreductase to nucleotidyltransferase, which is consistent with previous findings. Preference towards pigs appears to depend primarily on transferase activity and ion binding. Human-associated specificity factors incorporated loci coding for proteins participating in carbohydrate binding and the regulation of transcription (Figure 5b). The cellular localization of specificity factors associated with animal sources remained unchanged, yet more common functional terms were obtained (Figure 5c). However, it is worth highlighting that the cellular localization of specificity determinants associated with human hosts resembled avian-related ones, including the plasma membrane, cell periphery, and envelope.

Having described the main functional hallmarks of predicted specificity determinants, we utilized the k-means clustering method to group and explore the similarity between functional characteristics of factors linked with adaptation to certain hosts using an expanded dataset of associations according to raw *p*-values. The two metrics used (Shimkevich–Simpson and Jaccard coefficients) led to identical clustering patterns. While sets of enrichments related to avian, swine, and human hosts fell into one cluster, functional annotation terms within cattle-associated specificity factors formed a separate cluster (Figures S2 and S3).

## 3. Discussion

In the current research, we conducted a pangenomic assay using a curated dataset of 300 complete genome assemblies of *S. enterica* to uncover potential specificity factors associated with host–pathogen interactions. By examining the pangenome coupled with metadata of hosts affected, we identified potential host specificity determinants associated with the infection of birds, swine, and cattle. The accessory component of the reconstructed pangenome accounts for more than 90% of orthologous clusters. The obtained observation is consistent with previous studies in which similar percentages were reported [29,37,38]. Quite expectedly, the alpha parameter calculated using Heaps’ law being equal to 0.56 allows us to consider the *S. enterica* pangenome open (Figure 2a). Despite the first pangenomic study of 35 genomes conducted in 2011 defining the *Salmonella* pan-genome to be closed in contrast to *E. coli* [39], later analyses yielded comparable estimates to the one revealed by us, with an alpha parameter ranging from 0.3 to 0.6 [40,41]. It is noteworthy that even the usage of a single serovar confirmed the openness of the *S. enterica* pangenome [41,42]. Surprisingly, while the overall distribution of accessory genes clearly points to an open pangenome, individual serovars infecting a wide range of hosts may possess a closed one [37], which might be reflected by the significantly different fraction of core genes within genome groups isolated from different sources (Figure 2c). Notably, phylogenetic inferences have not constituted obvious compact clades with isolates infecting the same host, and an interspersed distribution of hosts along the tree was obtained instead (Figure 3). This result is consistent with other studies. Fenske et al. showed that the clustering of various *Salmonella* genomes corresponds to geographical origin rather than host specificity [28]. Even within a single serovar, Mbandaka genetic lineages infecting poultry and bovine hosts were scattered along the core SNP-based phylogeny [43]. No evidence of genomic differentiation between strains of *Salmonella enterica* serovar Typhimurium DT160 reflecting the shape of a phylogenetic tree was reported either [44].

We found a total of 237 positive associations within strains affecting distinct hosts (Table S5). Unlike those isolated from animal sources, there were no meaningful genomic features associated with human-infecting isolates. Such an observation might be explained by collapsing host-restricted serovars Typhi and Paratyphi into single reference clusters (Tables S2 and S3) and the prevalence of generalist strains. Moreover, when local outbreaks of enteric diseases occur, respective epidemiological studies deposit *Salmonella* genomes of unknown specificity, but attributed to human hosts.

Major specificity determinants that favor avian hosts as a preference fall into three categories: infectious process, antibiotic resistance, and transmission of genetic material. A strong association with a two-pore potassium channel plays its role in bacterial survival and invasion by both sustaining intracellular homeostasis and modulating the secretion of effectors by type III secretion systems [45]. The lamda phage-encoded CIII protein serves as an inhibitor of the FtsH protease, thereby assisting the phage in its propagation during the lytic lifecycle [46]. At the same time, FtsH proteolytic activity may also hydrolyze the MgtC virulence protein essential for successful proliferation in macrophages [47]; therefore, the inhibition of FtsH could promote the normal course of systemic infections. Another potential protective factor required for persistence is the Rhs protein, increasing the growth rate in macrophages [48]. We also revealed components of metabolic and transcriptional machinery enabling defense from antibiotics. One of them was TetC, which alters the transcriptome when *Salmonella* is exposed to tetracycline by regulating the transcription of the tetracycline efflux pump-encoding *tetA* gene and upregulating heat shock regulon *ibpAB* [49]. Two other determinants pose a threat to global health, being responsible for developing multidrug resistance (MDR), representing proteins with a broad spectrum of activities. These include multifunctional efflux protein MFS (major facilitator), excreting toxic metabolites from the cell [50], and ribokinase, capable of neutralizing antibiotics of variable chemical classes via phosphorylation [51]. Virulence and antibiotic-resistance genes frequently reside in mobile genetic elements (MGEs) and plasmids; therefore, their transmission might provide fitness to the hosts’ environment. The toxin/antitoxin CcdB/CcdA system maintains virulence plasmids’ stability, facilitating their intra- and inter-population flow. Of note, the respective genes are commonly observed in broad-host-range animal-infecting strains, but not in human-restricted serovars [52]. Tyrosine-dependent site-specific recombinase, in its turn, carries out the transfer of MGEs, with genes coding for distinct isoforms being related to host specificity in pathogenic bacteria [53]. The biological role of predicted determinants is backed by the respective functional annotation patterns. A variety of enrichments linked to ion binding and transport arose from the high prevalence of a two-pore potassium channel-encoding gene within the analyzed set of genomes (Figure 5b). The presence of various defense systems corresponded to terms indicating response to stress and chemicals accordingly (Figure 4a), whilst cellular localization assumes that the respective specificity factors reside primarily on the membrane (Figure 4c).

Strains isolated from pigs were enriched with genes encoding glycoside hydrolase and lysozyme, implying their involvement in the degradation of extracellular matrix and cell wall penetration, respectively, therefore facilitating colonization [54]. Subsequent infection steps, i.e., invasion of host cells and triggering inflammation response, are regulated by another identified determinant, guanine nucleotide exchange factor SopE, released through the type III secretion system [55]. At a later stage, improved intracellular survival is enhanced if the pathogen acquires the ability to exploit organic compounds synthesized by the host. For instance, ethanolamine is a prospective carbon and nitrogen source if metabolized by ethanolamine utilization protein EutE [56], associated with pig-infecting strains. The detected LexA family transcriptional regulator controls the expression of genes within the SOS regulon responsible for the general response of bacterial cells to environmental stresses, including antibiotics [57]. This suggests that certain homologs of the LexA-encoding loci might be attuned to the efficient prevention of stress occurring in particular hosts. Plenty of signals were given by genes localized in the transfer operon (*tra*), including *traM*, *traN*, *traL*, *traO*, etc. Insofar as the operon resides in the virulence plasmid and influences its dissemination [54], the gain or loss of these plasmids suggests alterations in host specificity. The acquisition of *tra*-containing plasmids shaped the evolution of *Yersinia pestis* [58] and *Klebsiella* sp. [59] through the emergence of distinct pathotypes ranging in virulence. Notably, we found plasmid stability genes, *psiB* and *psiA*, reported to be located in plasmids harboring prominent virulence factors as well [60]. Not unlike the genomic signatures of strains from avian sources, genomes of pig-specific isolates contained diverse agents of MGEs propagation, such as transposases belonging to the IS4 family and components of the bacteriophage integration. Transmissible insertions and prophages are known to house antibiotic-resistance genes and virulence loci [61,62]. The over-representation test indicated the involvement of specificity determinants in the detoxification of oxidative agents, as well as in catalytic activities, corroborating the detection of the SOS system’s transcriptional regulator, hydrolases, and the ethanolamine utilization protein, respectively (Figure 5a,b). According to the top GO enrichments from the CC ontology, the predicted determinants represent membrane and cytoplasmic proteins (Figure 5c).

Similar to the two above-mentioned groups, determinants of the preference for cattle as a host included those involved in pathogenesis, genetic exchange, and adaptation to stress. We found the Hha transcriptional factor was responsible for activating the expression of genes encoding hemolysins, pore-forming proteins easing the invasion of eukaryotic cells [63]. Upon entering host cells, a predicted Fe^3+^-siderophore permease could perform the import of iron ions into *S. enterica* cells. Enteric pathogens possess a wide range of iron-transporting proteins due to their essentiality for metabolic pathways, including those counteracting oxidative stress induced by the host immune system [64]. On top of this, we found disulfide isomerase DsbC constituting another strategy to detoxify reactive oxygen species through a reduction in the periplasmic copper-binding protein CueP [65]. Among the antibiotic resistance mechanisms, we identified the *floR* gene, which decreases the susceptibility to chloramphenicol via efficient efflux [66]. Additionally, we observed a bunch of signals of mercury resistance genes: *merD*, *merB*, and *merP*. The presence of these genes, possibly providing resistance to biocides, was reported in *S. enterica* serovars isolated from diseased animals [67]. The type IV secretion system VirD2 protein is involved in conjugative DNA transfer in enterobacteria-translocating genes responsible for adaptive traits [68]. The RecBCD nuclease inhibitor (Anti-RecBCD) usually impairs the restriction of foreign DNA, thus defending phages during integration [69]. This phage-encoded gene, though, resides in the multidrug-resistant *S.* Typhimurium ST313 strain, probably enhancing the dissemination of genetic islands [70]. We detected a bunch of DNA metabolism-related terms according to GO terms within molecular function ontology, implying the involvement of cattle-associated specificity factors in the intensive exchange of genetic loci through MGEs transmission (Figure 4b). Of note, the functional annotation of cellular localization resulted in significant enrichments with GO terms corresponding to encapsulating external structures apart from membrane proteins, thereby suggesting the importance of outer contact with host cells (Figure 4c). In summary, we showed that well-studied virulence determinants, including type III secretion systems, SopE, and antibiotic resistance factors, coupled with putative previously unreported candidates, appear to be associated with host specificity. The latter group is represented by a two-pore potassium channel, LexA family transcriptional regulators, and disulfide isomerase DsbC, hence their role in host restriction deserves further experimental validation.

Functional characteristics of virulence factors from VFDB encoded by genes the across studied genomic datasets generally resembled those obtained for putative specificity determinants. However, they predominantly centered around only one of three revealed categories: pathogenesis-related proteins. Top enrichments included transport, transcriptional regulation, and channel activity, i.e., typical processes taking place during infection stages (Figure S1). Resistance to environment stresses and the transmission of MGEs, contrarily, seemed to be linked with host specificity accordingly. Despite the functional similarity, a negligible fraction of specificity factors were homologs from VFDB, even though therew was a non-strict identity threshold. This suggests that the registry of known virulence factors appears to be incomplete, and *S. enterica* genomes harbor a wide range of genes encoding components of unexplored infection routes. On the other hand, the relationship between specificity (the ability to colonize a certain host) and virulence (the magnitude of pathological symptoms) might not be as straightforward as one might expect.

Pangenome-wide studies aimed at identifying genomic loci determining adaptation to hosts made on the basis of one, several, or multiple serovars provided similar findings with factors involved in metabolic adjustments, cell-to-cell contact, resistance to environment stress, and MGEs detected. For example, multi-host-adapted *S.* Enteritidis possessed frameshift mutations in the *sthC* gene encoding fimbrial outer membrane usher protein [27]. Parallel to this, preference towards mammalian hosts in *S.* Dublin was accompanied by improved glutamate metabolism, possibly alleviating intracellular growth, whereas in bird-restricted *S.* Pullorum, selection-driven mutations in the *ileS* gene possibly providing resistance to mupirocin were found [27]. Seif et al. detected unique altered catabolic pathways that increase the adaptation to hosts when infecting pigs, cattle, and chicken, including enzymes for utilizing D-tagatose, L-xylulose, D-xylose, deoxy-D-ribose, lidonate, D-glyceraldehyde, and allantoin. The traits mentioned were absent in strains isolated from swine and humans, suggesting the host diet contributes to the composition of metabolites in the intestinal tract, exerting an effect on *Salmonella* adaptation [37]. Similar findings were reported when analyzing strains from different animal sources [29]. Specific genetic signatures associated with avian sources involved genes encoding transporters, transcriptional regulators, RecD-like DNA helicase, and ketol-acid reductoisomerase [29]. Bowine-infected strains possessed unique accessory gene clusters coding for antitoxin protein HicB, phosphoethanolamine transferase EptC, and the replication-initiation protein RepE. Finally, genetic traits providing adaptation to a swine source constituted missense mutations in *iroN* (TonB-dependent siderophore receptor protein), and pepE (dipeptidase E)-modulating iron acquisition and aspartate synthesis, respectively [29]. The analysis of *S*. Mbandaka ST143 strains, which was focused on identifying the origin of the isolates for the sake of better epidemiological surveillance, revealed no universal pattern of specialization to bovine or avian hosts, but showed a variety of clonal populations forming distinct clades [43]. Groups of strains classified according to attributed hosts differed primarily in the composition of prophages; however, specific alleles in secretion systems were found as well [43]. Therefore, our results coupled with the existing pangenome-driven evidence show that not only does host specificity not imply a single evolutionary adaptation pathway, but also, in each particular case, it relies on diverse infection stages, pathogenic mechanisms, and genetic exchange, with different types of MGEs being involved.

## 4. Materials and Methods

### 4.1. Environments

Programming languages Python v.3.10.0 and R v.4.3.0 were used for data analysis. The results were visualized via the seaborn v0.12.2 [71] library and ggplot2 v.3.4.1 [72] package, respectively.

### 4.2. Data Acquisition

We utilized the Genome Updater (https://github.com/pirovc/genome_updater) (accessed on 3 July 2021) to download the genome assemblies of *Salmonella enterica* from the NCBI RefSeq database [30] with “complete genome” or “chromosome” assembly levels. To reduce possible false positive results in the pangenome analysis, assemblies with excessively high similarity were clustered using Assembly-Dereplicator (https://github.com/rrwick/Assembly-Dereplicator) (accessed on 2 November 2021) with a 99% identity and a sketch size of 100,000. Reference genomes were then selected from the resulting clusters for further research. The metadata of the assemblies, including the number of contigs and CDS (coding sequence), and genome size, were obtained using the Python script, which uses the Entrez Direct utility [73].

### 4.3. Pangenome Reconstruction

Panaroo v.1.3.0 [74] was used to reconstruct the pangenome. The tool was chosen for being more efficient in pangenome reconstruction. The underlying graphic representation of the pangenome allows for the imputting of missed genes in contrast to similarity-based methods. Moreover, the implemented algorithm sufficiently reduces the inflation of the accessory component, thus providing better clusterization patterns. The run was done with default settings, a 99% identity threshold for core gene identification, and in the “--remove-invalid-genes” mode. Core gene alignment was performed with MAFFT v.7 [75]. To assess the openness of the pangenome, the presence/absence tables were converted to binary matrices, and the micropan package v.2.1 [76] was used to calculate the alpha parameter based on Heaps’ Law. In addition, a power curve was plotted using a custom R script implementing the ggplot2 with 1000 permutations to calculate the number of genes after expanding the pangenome with new assemblies. The U-curve was built using a custom Python script implementing the seaborn library. Metadata of affected hosts were downloaded from the BioSample database [33] and PATRIC resource [32] using a custom Python script. The percentage of core genome per host was calculated as the division accessory component and core component of pangenome and visualized using Python script and seaborn library. The groups were then compared with a pairwise Wilcoxon test corrected for multiple comparisons using the Benjamini–Hochberg (BH) procedure.

### 4.4. Phylogenetic Analysis

We applied two different methods to reconstruct the phylogeny, considering both recombination-prone and non-recombination-prone core genes. To identify regions that have undergone recombination, we used the ClonalFrameML v.1.12 tool [77] based on the concatenated alignment of core genes cleaned from ambiguous bases via replacing them with the most frequent bases and guiding phylogenetic tree building with the FastTree v.2 program [78]. We then extracted core SNPs (Single Nucleotide Polymorphism) utilizing the SNP-sites v2.5.1 tool [79], and either removed recombination signals or retained them. The best-fit evolutionary models were chosen using ModelTest-NG v0.1.7 [80] based on the Bayesian Information Criterion (BIC). The RAxML-NG v1.1.0 [81] program was used to reconstruct maximum likelihood (ML) phylogenetic trees with 1000 bootstrap replications, employing the optimal model selected earlier. Reference phylogeny coupled with a heatmap colored according to affected hosts and serovars was visualized with the ggtree v.3.8.0 library [82]. The reconstructed trees were topologically compared using the tqDist v1.0.2 library [83] with quartet distance metrics and the Robinson–Fuld metric applying the RAxML-NG tool, respectively. The quality of the reconstructed trees was assessed based on two metrics, namely, consistency index (CI) and retention index (RI), calculated in the phangorn v.2.11.1 package [84]. Additionally, we calculated the Colles–Like balance index using the CollesLike v2.0 package [85].

### 4.5. Virulence and Specificity Factors Analysis

We used the above-described metadata for attributing reference genomes to infected hosts, considering all groups of hosts, referring to the strains within each cluster to retain information for further association analysis by applying a custom Python script implementing the pandas v.2.0.1 library. To ensure enough statistical power, we discarded hosts associated with fewer than 10 assemblies. Next, gene presence/absence distribution and binary table with host attributions were used to identify candidate genes associated with host specificity using pyseer v1.3.5 [35]. We then considered positive associations (beta > 0) and negative (beta < 0) associations passing all the filters, i.e., removed inferences marked with “bad” Chi-Square test results and insignificant singles. Two datasets were further analyzed: significant according to BH-corrected and raw *p*-values. We also searched for known virulence factors deposited in the VFDB (virulence factor database) [86] using the MMseqs2 v.14.7 tool [87]. The presence of homologs was considered if the hists showed identity and mutual coverage of at least 70%. Next, the percentage of such homologs was calculated within the sets of pyseer-predicted gene sets.

### 4.6. Functional Annotation

The functional annotation of protein sequences within pangenomic clusters was carried out using the eggNOG v2.0.1b-2-g816e190 tool in the “mmseqs2” [88] search mode within the GO (Gene Ontology) annotation system [89]. The over-representation test was performed using the topGO v.3.15 package [90]. Significant enrichments according to the FDR (false detection rate) adjustment were selected. We then compared functional annotation patterns within host-wise groups of specificity determinants. To achieve this, a k-means clustering algorithm was applied to the distance matrix implementing Shimkevich–Simpson and Jaccard coefficients using the Python script implementing scikit-learn v.1.1 and pandas v.2.0.1 libraries. The optimal number of clusters was determined by silhouette analysis using the R script implementing the factoextra v.1.0.7 package [91]. The clustering results were subsequently visualized using the “autoplot” function from ggfortify v0.4.11 [92].

## 5. Conclusions

Being able to infect a wide range of mostly warm-blooded vertebrates, *S. enterica* represents a universal pathogen posing a threat to both public health and agriculture. Given the great divergence of distinct serovars varying from generalists to specialists in terms of host specificity, the species has become a convenient and extensively studied model of host specificity mechanisms. To the best of our knowledge, the current research is the first pangenomic study to reveal specificity determinants of *S. enterica* without the preliminary selection of certain serovars or available datasets. In our research, we carried out a data-driven search of genes associated with host restriction. The complexity of the task lies in the sparseness of metadata regarding the host specificity of the isolates analyzed. That being said, the absence of experimentally validated inferences related to the ability and, arguably more crucial, inability to infect certain hosts might spark unavoidable artefactual results. We, however, identified sets of genes, in which absence and presence patterns appear to delineate host preference. We suggested categorizing revealed specificity factors into three main groups pertaining to the biological processes they participate in, namely, pathogenesis, resistance to antibiotics, and the propagation of mobile genetic elements (MGEs). It is worth noting that we have not found associations between affected host species and known virulence determinants, which are shown to be directly involved in the invasion of host cells. We conclude that the presence of certain genes might facilitate the adaptation to the environment or affect genome stability, whereas loci encoding invasion systems are always present in the genome while differing within single nucleotide polymorphic sites. Therefore, the pangenome-wide data-driven approach chosen is applicable for mining genes associated with adaptation to the environment of the new host, since a large fraction of those found fit in with existing evidence obtained independently by other researchers.

## Data Availability

All scripts used in this work are available at https://github.com/lab7arriam/salmonella_pangenome (the latest commit on 26 August 2023). All data are available as Supplementary Materials.

## Supplementary Materials

The following supporting information can be downloaded at: https://www.mdpi.com/article/10.3390/ijms241813670/s1.

## Abbreviations

MGEs	Mobile genetic elements
CDC	Centers for Disease Control and Prevention
LPS	Lipopolysaccharides
T3SS	Type III secretion systems
SCV	*Salmonella*-containing vacuole
tcf	Typhi colonization factor
BH	Benjamini-Hochberg
SNP	Single nucleotide polymorphism
BIC	Bayesian information criterion
CI	Consistency index
RI	Retention index
VFDB	Virulence factor database
GO	Gene Ontology
MF	Molecular Functions
BP	Biological Processes
CC	Cellular Components
pan-GWAS	Pangenome-wide associating method
MDR	Multidrug resistance
MFS	Major facilitator
*tra*	Transfer operon
