# Supplementary material for: For Someone, You Are the Whole World: Host-Specificity of Salmonella enterica
Source: Int J Mol Sci. 2023 Sep 5;24(18):13670. doi: 10.3390/ijms241813670 (PMC10530738; doi:10.3390/ijms241813670)
Supplement: Supplementary file 1 [file ijms-24-13670-s001.zip › Supplementary_figures.pdf]

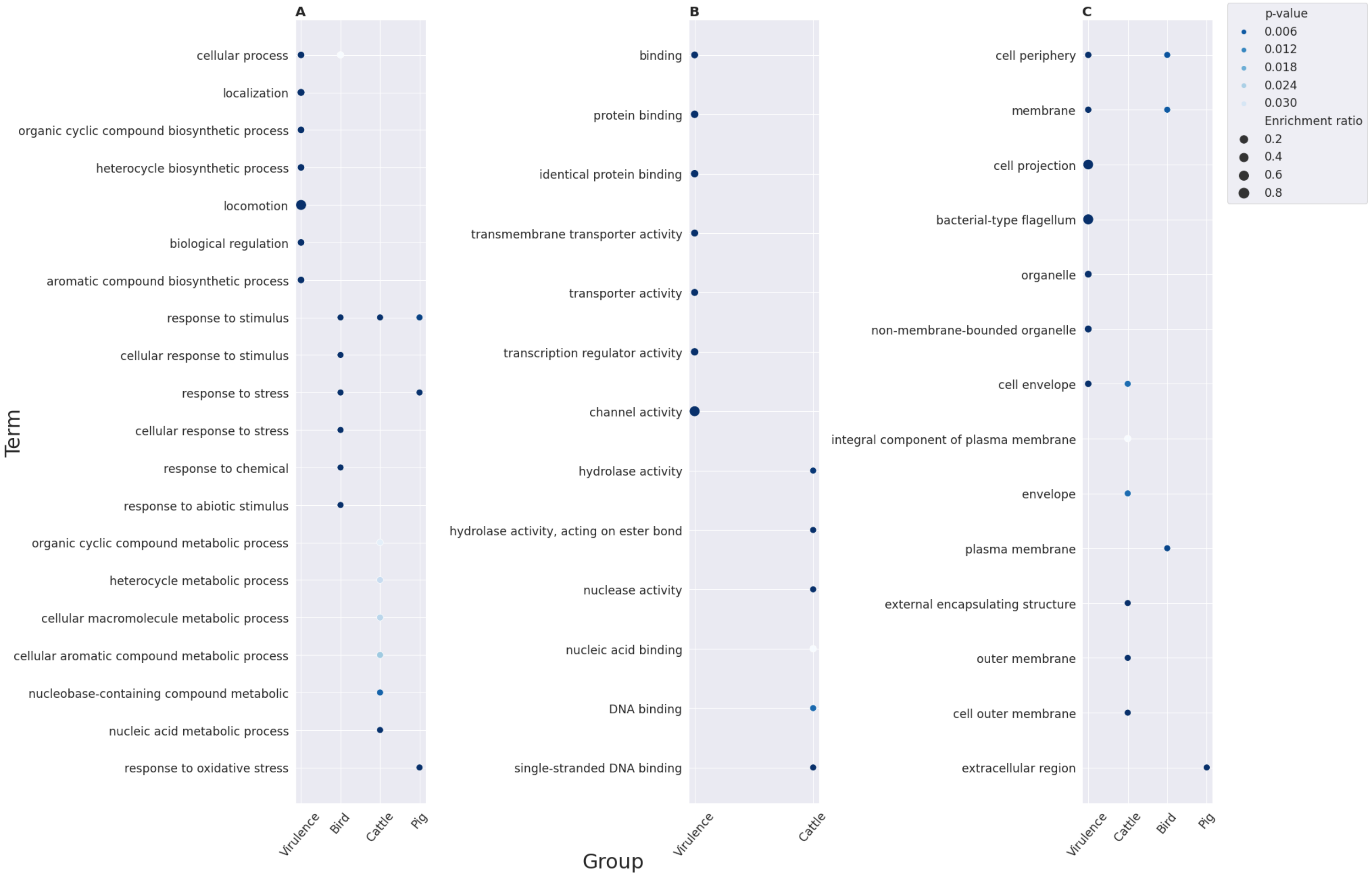

Figure S1 Functional annotation of host specificity factors with over-represented terms the using GO (Gene Ontology) annotation system with virulence factor. Shown are the results for the sets of pyseer-reported positive associations with certain hosts filtered according to the Benjamini-Hochberg (BH) procedure correction for multiple comparisons within the Biological processes (**A**), Molecular functions (**B**), and Cellular components (**C**) ontologies. The dot size depicts the enrichment ratio, while the intensity of the color is proportional to p-values.

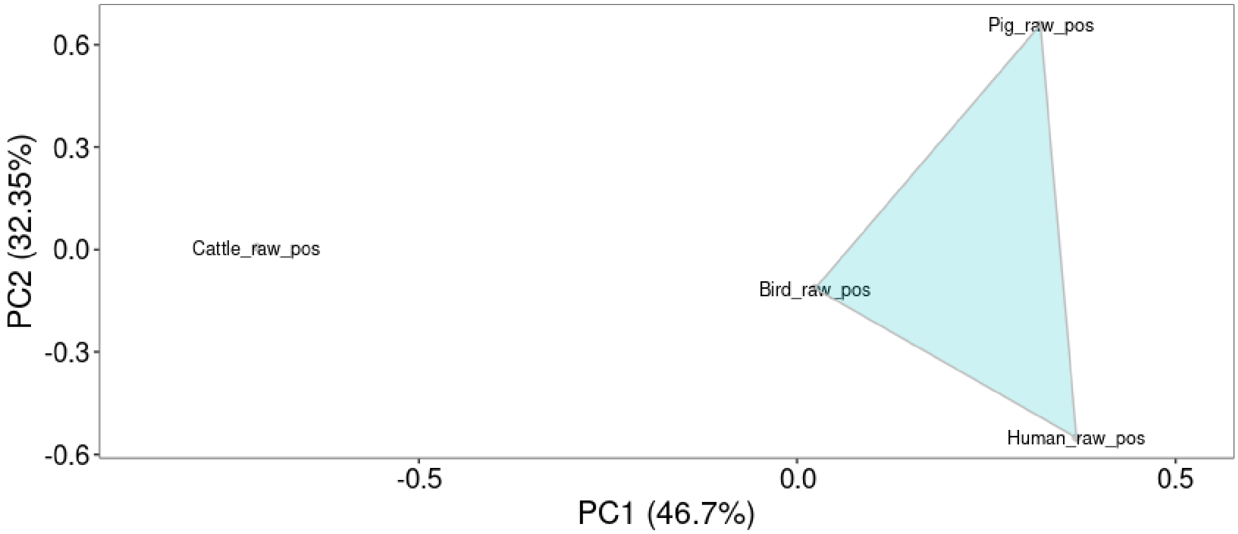

Figure S2. The k-means algorithm clustering results using Shimkevich–Simpson metrics based on significant functional annotation enrichments within groups attributed to different hosts.

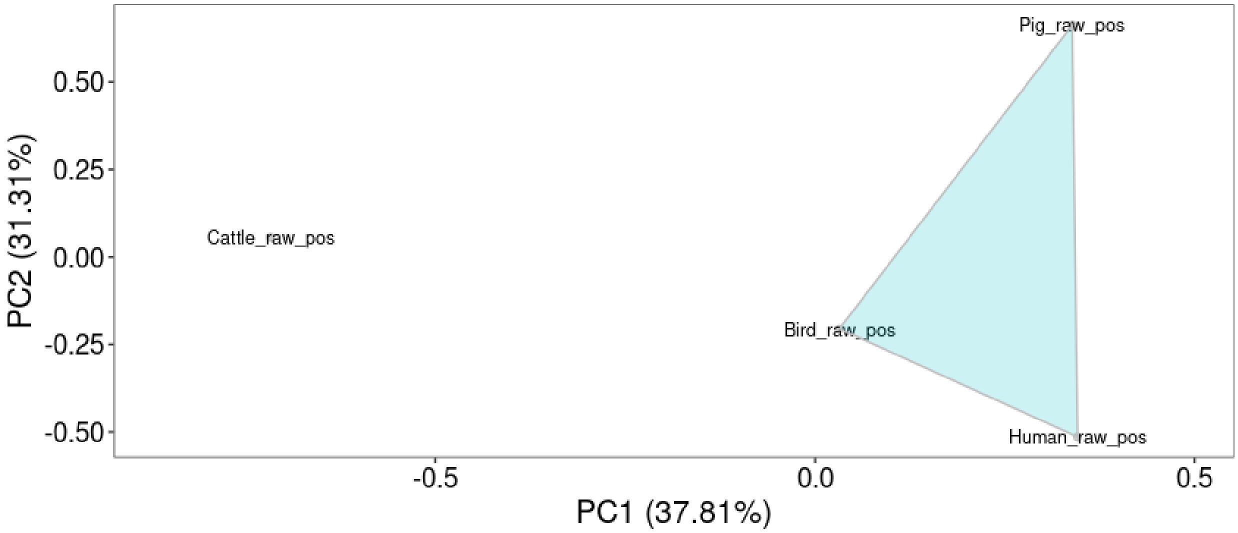

Figure S3. The k-means algorithm clustering using Jaccard coefficients metrics results based on significant functional annotation enrichments within groups attributed to different hosts.
